# Supplementary material for: An unexpected INAD PDZ tandem-mediated plcβ binding in Drosophila photo receptors
Source: eLife. 2018 Dec 10;7:e41848. doi: 10.7554/eLife.41848 (PMC6300352; doi:10.7554/eLife.41848)
Supplement: Supplementary file 1: [file elife-41848-supp1.docx]

**Supplementary file 1: Statistics of data collection and model refinement for NORPA CC-PBM structures, INAD/NORPA complex structure and INADL/PLCβ4 complex structure.**

| **Data collection** | | | | |
| --- | --- | --- | --- | --- |
| Data sets | Se-Met NORPA CC-PBM K884E K898E | methylated NORPA CC-PBM | NORPA CC-PBM^8KA^/INAD PDZ45 | Gold-derived PLCβ4 CC-PBM^8KA^/INADL PDZ89 |
| Space group | *P1* | *P6_1_22* | *C222_1_* | *P3_1_21* |
| Wavelength (Å) | 0.9792 | 0.9793 | 0.9780 | 0.9792 |
| Unit cell parameters (Å) | a=37.0, b=37.6, c=140.1,  α= 90.7°, β=89.9°, γ=119.4° | a=b=119.2, c=116.1,  α=β=90°, γ=120° | a=117.946, b=157.213,  c=60.986,  α=β=γ=90° | a=b=69.089, c=200.375,  α=β=90°, γ=120° |
| Resolution range (Å) | 50-2.70  (2.75-2.70) | 50-3.50  (3.56-3.50) | 50-3.25  (3.31-3.25) | 50-2.80  (2.85-2.80) |
| No. of unique reflections | 17843 (834) | 5961 (300) | 9527 (475) | 14070 (710) |
| Redundancy | 3.4 (3.3) | 11.4 (11.4) | 5.9 (5.9) | 7.2 (6.9) |
| I/σ | 21.3 (1.7) | 21.8 (2.9) | 25.1 (2.4) | 26.2 (2.2) |
| Completeness (%) | 96.6 (95.4) | 94.4 (94.9) | 99.6 (100) | 99.7 (99.6) |
| R_merge_ ^a^ (%) | 10.8 (98.4) | 13.3 (64.8) | 8.3 (85.4) | 11.7 (93.8) |
| CC_1/2_ for highest resolution shell | 0.785 | 0.702 | 0.622 | 0.954 |
| **Structure refinement** | | | | |
| Resolution (Å) | 50-2.70  (2.84-2.70) | 50-3.50  (4.45-3.50) | 50-3.25  (3.72-3.25) | 50-2.80  (3.09-2.80)* |
| R_cryst_ ^b^/R_free_ ^c^ (%) | 22.76/27.98  (30.52/32.92) | 24.67/29.55  (28.68/36.75) | 22.78/27.52  (25.84/35.66) | 24.68/30.22  (31.53/32.87) |
| Rmsd bonds (Å) / angles (°) | 0.007/1.150 | 0.007/0.955 | 0.008/1.002 | 0.008/1.192 |
| Average B factor ^d^ | 94.2 | 103.7 | 119.2 | 28.9 |
| No. of atoms | | | | |
| Protein atoms | 3357 | 1521 | 2981 | 3189 |
| Water | 0 | 0 | 1 | 0 |
| Other molecules | 4 | 0 | 0 | 5 |
| No. of reflections | | | | |
| Working set | 16719 (2677) | 5619 (2716) | 8745 (2857) | 11926 (1776) |
| Test set | 887 (145) | 319 (157) | 474 (168) | 613 (110) |
| Ramachandran plot regions ^d^ | | | | |
| Favored (%) | 96.8 | 97.0 | 96.3 | 97.9 |
| Allowed (%) | 3.2 | 3.0 | 3.7 | 2.1 |
| Outliers (%) | 0.3 | 0 | 0 | 0 |

Numbers in parentheses represent the value for the highest resolution shell.

a. R_merge_ = Σ |*I_i_* - <*I*>| / Σ*I_i_*, where *I_i_* is the intensity of measured reflection and <*I*> is the mean intensity of all symmetry-related reflections.

b. R_cryst_=Σ||*F*_calc_| – |*F*_obs_||/Σ*F*_obs_, where *F*_obs_ and *F*_calc_ are observed and calculated structure factors.

c. R_free_= Σ_T_||*F*_calc_| – |*F*_obs_||/Σ*F*_obs_, where T is a test data set of about 5% or 10% of the total unique reflections randomly chosen and set aside prior to refinement.

d. B factors and Ramachandran plot statistics are calculated using MOLPROBITY.

*: The data were corrected for anisotropy with truncations to 3.0 Å, 3.1 Å and 2.8 Å along the a, b and c axes, respectively.
